# Laterality of specific binding ratios on DAT-SPECT for differential diagnosis of degenerative parkinsonian syndromes

**DOI:** 10.1038/s41598-020-72321-y

**Published:** 2020-09-25

**Authors:** Taro Shigekiyo, Shigeki Arawaka

**Affiliations:** Division of Neurology, Department of Internal Medicine IV, Osaka Medical College, 2-7 Daigaku-machi, Takatsuki, Osaka 569-8686 Japan

**Keywords:** Neurology, Neurological disorders, Parkinson's disease

## Abstract

Motor symptoms of Parkinson’s disease (PD) occur unilaterally and progress with asymmetry, while progressive supranuclear palsy (PSP) and multiple system atrophy of the parkinsonism subtype (MSA-P) lack this tendency. We assessed the laterality of specific binding ratios (SBRs) on dopamine transporter single-photon emission computed tomography (DAT-SPECT) for the differential diagnosis of these diseases in 311 PD, 33 PSP, 20 MSA-P, and 137 control patients. The average SBR in PD was higher than that in PSP (P = 0.035). Compared with Hoehn–Yahr (HY) stages, the average SBR in PD with HY stage I was only higher than that in PSP (P < 0.001). SBR laterality in PD with HY stage I was significantly higher than that in PSP (P = 0.001). This difference was not observed in PD with HY stage II. The average and laterality of SBRs in MSA-P were similar to those in PD and PSP. The asymmetry indices were similar among PD, PSP, and MSA-P. These data suggest that PSP shows a pattern of SBRs different from that in PD, attributed to HY stage I in PD. The limited usefulness of DAT-SPECT may be explained by the low discrimination between PD with bilateral motor symptoms and PSP.

## Introduction

According to the UK Parkinson’s Disease Society Brain Bank clinical diagnostic criteria for Parkinson’s disease (PD), parkinsonism is defined as any one of muscle rigidity, 4–6 Hz rest tremor, and postural instability, in addition to bradykinesia^1^. The diagnosis of definite PD requires three or more of the following items: unilateral onset, rest tremor, progressive course, persistent asymmetry affecting the side of onset, excellent response to levodopa, severe levodopa-induced dyskinesia, levodopa response for more than 5 years, or a clinical course of more than 10 years. As indicated in these diagnostic criteria, the motor symptoms of PD are clinically characterised by asymmetry of the affected limbs observed at any time. Among the Movement Disorder Society’s (MDS) clinical diagnostic criteria, bilateral symmetric parkinsonism is a relative exclusion criterion^2^. To differentiate PD from progressive supranuclear palsy (PSP) and multiple system atrophy of the parkinsonism subtype (MSA-P), it is important to confirm whether there is persistent asymmetry in motor symptoms.

Dopamine transporter single-photon emission computed tomography (DAT-SPECT) with ^123^I‐Ioflupane is used to assess DAT function at the presynaptic nerve endings of nigrostriatal dopamine neurons^3,4^. On DAT-SPECT, DAT function is quantitatively assessed based on specific binding ratios (SBRs) of striatal ^123^I‐Ioflupane accumulation. According to the MDS clinical diagnostic criteria, a normal finding on DAT-SPECT is one of the absolute exclusion criteria for PD diagnosis^2^. The clinical utility of DAT-SPECT has been shown in differentiating PD from non-degenerative parkinsonism^5–8^. However, DAT-SPECT commonly shows a reduction in ^123^I‐Ioflupane accumulation in the striatum of PD, PSP, and MSA-P^9,10^. Previous studies have demonstrated that DAT-SPECT is not helpful for diagnosing these degenerative parkinsonian syndromes^11–13^. Pirker et al. demonstrated that asymmetry of striatal ^123^I‐Ioflupane binding seemed to be less pronounced in MSA and PSP than in PD with no statistical difference^14^. It is unclear how the asymmetry of motor symptoms in different stages of PD is reflected on DAT-SPECT imaging and whether asymmetric reduction in striatal ^123^I‐Ioflupane accumulation on DAT-SPECT is useful to differentiate PD from PSP and MSA-P. In this study, we assessed the average and laterality of SBRs on DAT-SPECT for the differential diagnosis of PD, PSP, and MSA-P.

## Results

### Average SBRs on DAT-SPECT in the PD, PSP, and MSA-P groups

We first compared the demographic data in the PD, PSP, MSA-P, and control groups (Table 1). The age at the time of DAT-SPECT imaging in the PD group was significantly lower than that in the control group (P = 0.001). There was no difference in the age at the time of DAT-SPECT imaging among the PSP, MSA-P, and control groups. Additionally, there was no difference in disease duration at the time of DAT-SPECT imaging among the PD, PSP, and MSA-P. The proportions of men were comparable among all groups.

**Table 1:** Summary of patient background of the PD, MSA-P, PSP and control groups.

	PD (n = 311)	PSP (n = 33)	MSA-P (n = 20)	Control (n = 137)
Age	73.0* (66.0–78.0)	74.0 (70.5–79.5)	71.5 (65.8–75.8)	76.0 (72.0–80.0)
Men, n (%)	158 (50.8)	18 (54.5)	12 (60.0)	62 (45.3)
Duration (y)	2.0 (1.0–3.0)	2.0 (1.0–2.0)	2.0 (2.0–4.5)	

	HY stages of PD			
	HY I (n = 88)	HY II (n = 106)	HY III (n = 89)	HY IV (n = 28)
Age	70.0 (63.0–76.0)	73.0 (66.0–77.3)	75.0 (66.5–78.5)	77.5^†^ (72.0–82.0)
Men, n (%)	38 (43.2)	57 (53.8)	53 (59.6)	10 (35.7)
Duration (y)	1.0 (1.0–2.0)	2.0 (1.0–3.0)	3.0^††^ (2.0–5.0)	4.5^††^ (2.0–7.5)

Data are expressed as a median and interquartile range (IQR) or n (%).

*PD* Parkinson’s disease, *PSP* progressive supranuclear palsy, *MSA-P* multiple system atrophy of the parkinsonism subtype, *HY* Hoehn-Yahr stage.

Duration shows years of DAT-SPECT imaging from onset. *indicates P < 0.01 as compared with the control group (SPSS software, version 23.0). ^†^indicates P < 0.01 and ^††^ indicates P < 0.001 as compared with the PD group with HY stage I.

**Figure 1:**
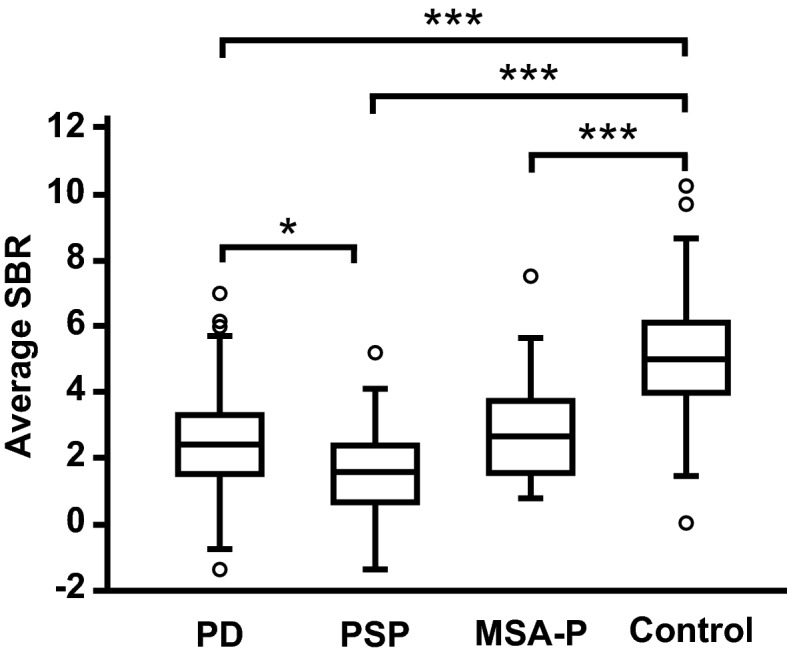
Comparison of average SBRs on DAT-SPECT in the PD, PSP, and MSA-P groups. Comparison of average SBRs among the PD, PSP, MSA-P, and control groups. The box-and-whisker plots show the median and interquartile range (box), the upper hinge (75th percentile plus 1.5 times the interquartile range) (upper error bars), the lower hinge (25th percentile minus 1.5 times the interquartile range) (lower error bars), and outlier values (circles) outside the hinges. The figure image was generated by Adobe Illustrator CC 2017 (ver. 21.1.0). Data were analysed by Kruskal–Wallis test with Bonferroni correction (SPSS software, version 23.0). *P < 0.05; ***P < 0.001.

To assess the clinical usefulness of DAT-SPECT in the diagnosis of degenerative parkinsonian syndromes, we compared the average SBRs in the PD, PSP, MSA-P, and control groups (Fig. 1). The median values of the average SBRs were 2.37 [interquartile range (IQR) 1.58–3.28] in the PD group, 1.55 (IQR 0.66–2.39) in the PSP group, and 2.64 (IQR 1.50–3.72) in the MSA-P group. The values of the average SBRs in these degenerative parkinsonian syndromes were significantly lower than the value in the control group (median 4.97; IQR 4.01–6.08), (P < 0.001 in each case). Additionally, the value of the average SBR in the PD group was significantly higher than that in the PSP group (P = 0.035). However, there was no difference in the values of the average SBRs between the PD and MSA-P groups (P = 1.000) and between the PSP and MSA-P groups (P = 0.109).

### Average SBRs on DAT-SPECT in the PD with different Hoehn-Yahr stages, PSP and MSA-P groups

As compared with the PD group with Hoehn-Yahr (HY) stage I, the age at the time of DAT-SPECT imaging was significantly higher in the PD group with HY stage IV (P = 0.002) (Table 1). Disease duration was significantly longer in the PD groups with HY stage III and IV (P < 0.001 in each case). The proportions of men were comparable among different HY stages.

**Figure 2:**
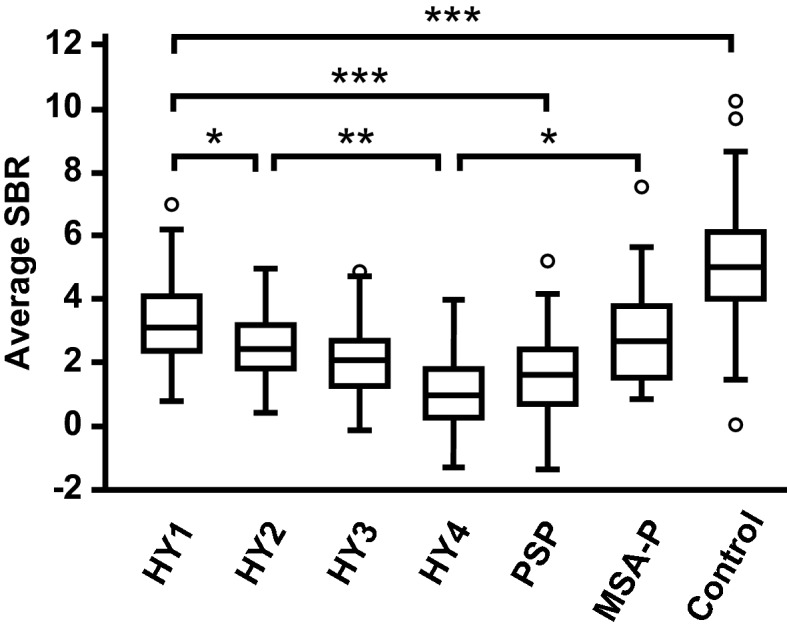
Comparison of average SBRs on DAT-SPECT in the PD with different Hoehn-Yahr stages, PSP, and MSA-P groups. Comparison of average SBRs among the PD with different Hoehn-Yahr (HY) stages, PSP, MSA-P, and control groups. The box-and-whisker plots show the median and interquartile range (box), the upper hinge (75th percentile plus 1.5 times the interquartile range) (upper error bars), the lower hinge (25th percentile minus 1.5 times the interquartile range) (lower error bars), and outlier values (circles) outside the hinges. The figure image was generated by Adobe Illustrator CC 2017 (ver. 21.1.0). Data were analysed by Kruskal–Wallis test with Bonferroni correction (SPSS software, version 23.0). *P < 0.05; **P < 0.01; ***P < 0.001.

When we assessed average SBRs in the different HY stages of PD, the values of the average SBRs decreased with progression in the HY stage (HY I vs. HY II, P = 0.039; HY II vs. HY IV, P = 0.002) (Fig. 2). To assess whether disease progress of PD affects the difference in the average SBRs between PD and PSP, we compared the average SBRs among the PD with different HY stages and PSP groups. The value of the average SBR in the PD group with HY stage I was significantly higher than that in the PSP group (P < 0.001) (Fig. 2). However, there was no significant difference in the average SBRs between the PD group with HY stage II or higher and the PSP group (HY II vs. PSP, P = 0.146; HY III vs. PSP, P = 1.000; HY IV vs. PSP, P = 1.000). Additionally, there was no difference in the values of the average SBRs between the PD group with HY stage I and MSA-P group (P = 1.000), while the value of the average SBR in the PD group with HY stage IV was significantly lower than that in the MSA-P group (P = 0.020).

### SBR laterality on DAT-SPECT in the PD, PSP, and MSA-P groups

**Figure 3:**
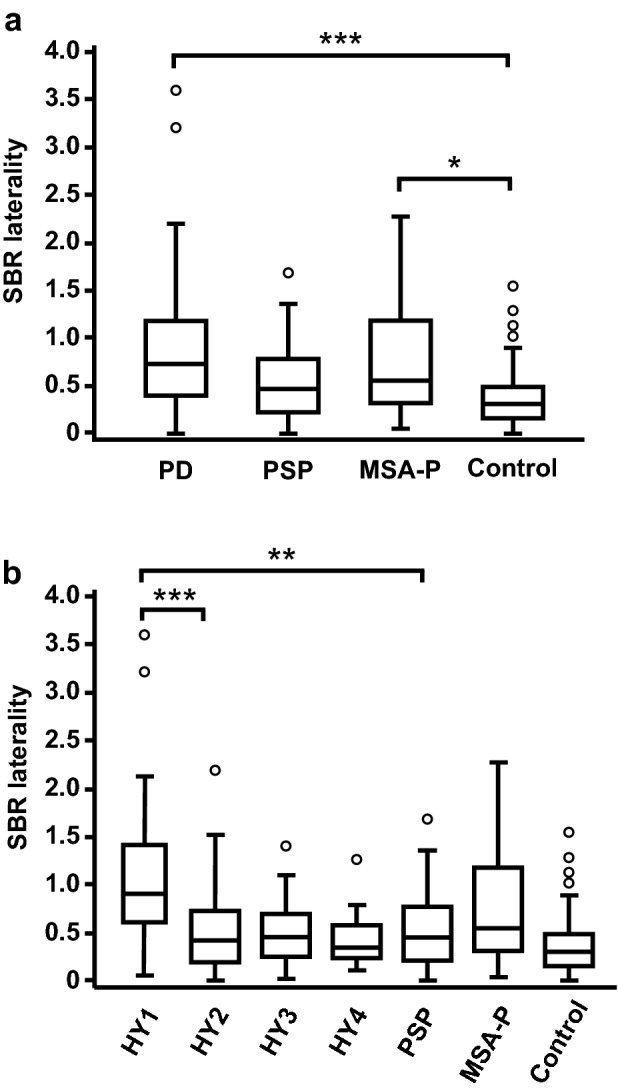
Comparison of SBR laterality in the PD, PSP, and MSA-P groups. (**a**) Comparison of SBR laterality among the PD, PSP, MSA-P, and control groups. (**b**) Comparison of SBR laterality among the PD with different HY stages, PSP, MSA-P, control groups. The box-and-whisker plots show the median and interquartile range (box), the upper hinge (75th percentile plus 1.5 times the interquartile range) (upper error bars), the lower hinge (25th percentile minus 1.5 times the interquartile range) (lower error bars), and outlier values (circles) outside the hinges. The figure images were generated by Adobe Illustrator CC 2017 (ver. 21.1.0). Data were analysed by Kruskal–Wallis test with Bonferroni correction (SPSS software, version 23.0). *P < 0.05; **P < 0.01; ***P < 0.001.

To assess the usefulness of SBR laterality on DAT-SPECT in the diagnosis of degenerative parkinsonian syndromes, we calculated the absolute values of SBR laterality by simply subtracting the SBR value of one side from that of the contralateral side. The values of SBR laterality were 0.53 (IQR 0.26–0.88) in the PD group, 0.44 (IQR 0.22–0.77) in the PSP group, 0.55 (IQR 0.31–1.17) in the MSA-P group, and 0.29 (IQR 0.16–0.48) in the control group (Fig. 3a). The values of SBR laterality in the PD and MSA-P groups were significantly higher than the value in the control group (PD vs. control, P < 0.001; MSA-P vs. control, P = 0.010). There was no difference in the values of SBR laterality between the PSP and control groups (P = 1.000). Additionally, the values of SBR laterality were comparable among the PD, PSP, and MSA-P groups (P = 1.000 in each case). When we assessed SBR laterality in the different HY stages of PD, the values of SBR laterality decreased between HY stages I and II (P < 0.001) (Fig. 3b). The value of SBR laterality in the PD group with HY stage I was significantly higher than that in the PSP group (P = 0.001). However, the difference in SBR laterality was not observed in the PD group with HY stage II or higher (HY II vs. PSP, HY III vs. PSP, HY IV vs. PSP, P = 1.000 in each case). There was no difference in the values of SBR laterality between the PD group with HY stage I and MSA-P group (P = 0.538).

### Asymmetry index values on DAT-SPECT in the PD, MSA-P, and PSP groups

**Figure 4:**
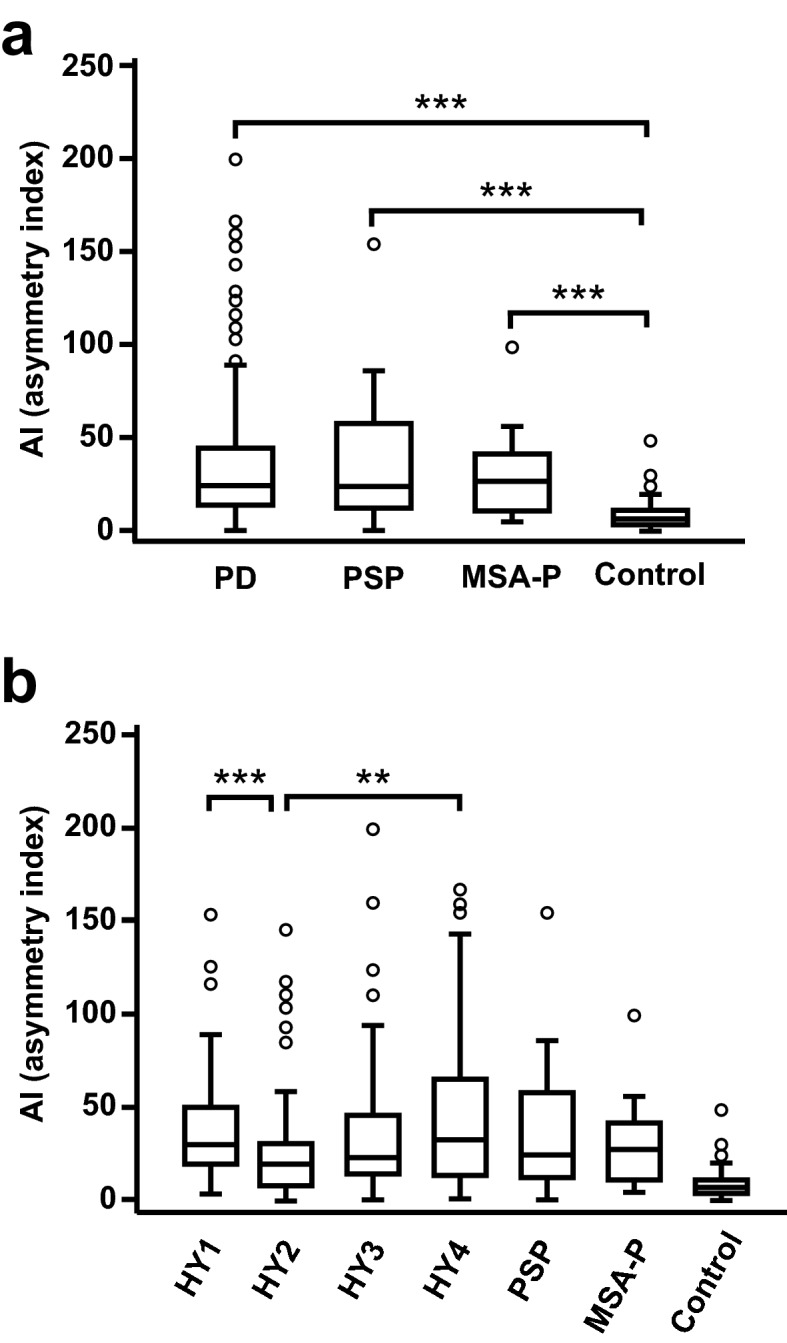
Comparison of AI values in the PD, PSP, and MSA-P groups. (**a**) Comparison of AI among the PD, PSP, MSA-P, and control groups. (**b**) Comparison of AI among the PD with different HY stages, PSP, MSA-P, and control groups. The box-and-whisker plots show the median and interquartile range (box), the upper hinge (75th percentile plus 1.5 times the interquartile range) (upper error bars), the lower hinge (25th percentile minus 1.5 times the interquartile range) (lower error bars), and outlier values (circles) outside the hinges. The figure images were generated by Adobe Illustrator CC 2017 (ver. 21.1.0). Data were analysed by Kruskal–Wallis test with Bonferroni correction (SPSS software, version 23.0). **P < 0.01; ***P < 0.001.

The asymmetry index (AI) is used as an indicator to assess the asymmetry of striatal ^123^I‐Ioflupane reduced accumulation on DAT-SPECT. We analysed the usefulness of the AI in the diagnosis of PD, MSA-P, and PSP (Fig. 4a). The AI values were 23.78 (IQR 12.99–43.97) in the PD group, 23.91 (IQR 11.20–57.63) in the PSP group, and 26.67 (IQR 9.93–41.33) in the MSA-P group. These AI values were significantly higher than the values in the control group (median 5.60; IQR 3.04–10.43) (P < 0.001 in each case). However, no significant difference was observed among the PD, MSA-P, and PSP groups (P = 1.000 in each case). When the AI was assessed in the different HY stages of PD, the values of the AI did not constantly show an decreasing tendency with progression in the HY stage (Fig. 4b). There were no differences in the values of the AI among the PD group with HY stage I, PSP group, and MSA-P group (P = 1.000 in each case).

## Discussion

A previous study showed that striatal ^123^I‐Ioflupane accumulation is reduced in PD, PSP, and MSA-P, and the striatal reduction is more significant in PSP with no statistical difference between that in PD and MSA-P^15,16^. In this study, we found that the average SBR in MSA-P showed no difference from that in PD and PSP. Additionally, we found that the average SBR in PSP is significantly lower than that in PD. On assessing the values of the average SBRs in different HY stages, there was a difference in the values between PSP and PD with HY stage I, but the difference was not observed in PD with HY stage II or higher. These findings suggest that the value of the average SBR in PD with HY stage I may affect the difference between PD and PSP because the number of patients with PD with HY stage I was much smaller than that of patients with PD with HY stages II to IV (88 with HY stage I vs. 223 with HY stages II-IV). In support of this idea, the value of SBR laterality in PD with HY stage I was only higher than that in PSP. These findings suggest that patients with unilateral parkinsonism have a high probability of PD or MSA-P on DAT-SPECT, and the SBRs on DAT-SPECT are not useful for the differential diagnosis of patients with bilateral parkinsonism. Low discrimination in patients with bilateral parkinsonism may yield a limited usefulness of DAT-SPECT in the differential diagnosis of PD and PSP.

In most patients with PD, there is a substantial asymmetry of clinical motor symptoms from disease onset. A previous pathological study of 21 patients with PD showed that there was greater loss of nigral neurons contralateral to the initially affected side^17^. However, it is unclear as to the mechanism generating asymmetry of clinical motor symptoms. One possibility is that the number of nigral neurons on one side is different from that on the other side from birth, and the side with the reduced number of neurons reaches the threshold of clinical manifestations earlier^18^. Second possibility is that nigral neurons on one side are more vulnerable than those on other side^18^. Once the degenerative process starts, cell death occurs on the vulnerable side earlier. The present data showed the value of SBR laterality in the control group was the lowest in the subject groups. This finding may support the latter possibility from the point of striatal ^123^I‐Ioflupane accumulation on DAT-SPECT. Additionally, the present data showed that the values of SBR laterality were significantly compressed from HY stage II. It raises an idea that nigrostriatal dopaminergic neurons contralateral to the initially unaffected side may accelerate degeneration in HY stage II. This idea may explain the difficulty of DAT-SPECT for the differential diagnosis of patients with bilateral parkinsonism.

The AI is commonly used as an indicator to evaluate SBR laterality^14^. However, the present data demonstrated that the values of the AI were not useful to differentiate PD with HY stage I from PSP, and they failed to show a decreasing tendency with progression of HY stage. In contrast, SBR laterality reflected progress of PD, because it matched with unilateral decrease of striatal ^123^I‐Ioflupane accumulation in HY stage I and loss of unilaterality in more advanced HY stages. This might be attributed to the definition of the AI. The value of the AI was calculated using the following formula: | SBRleft – SBRright | / [(SBRleft + SBRright)/2] × 100^7,19^. As the value of the SBR on one side approaches zero in the advanced stage, the value of the AI increases, generating variability in the values of the AI.

It should be noted that the age at the time of DAT-SPECT imaging in the PD group was significantly younger than that in the control group. Generally, the values of SBR tend to decrease with aging^20^. This finding suggests that the difference in average SBRs between the PD and control groups in this study might be smaller than that in the age-matched comparison. However, there was no significant difference in the age among the PD, PSP and MSA-P groups, showing that the age did not affect the comparison of SBR values among these diseases. In addition, the age at DAT-SPECT imaging in the PD groups with HY stage I was significantly younger than that in the PD group with HY stage IV. This may attenuate the difference in SBR values between HY stages I and IV. However, there was no difference in the age among the PD groups with HY stages I, II, and III, showing that the age did not affect the comparison of SBR values among these HY stages. There were several limitations of this study. The imaging system of DAT-SPECT used in this study did not set the region of interest at the subregional levels, such as caudate, anterior putamen, and posterior putamen, although subregional analysis may provide more detailed information regarding the clinical usefulness of DAT-SPECT. The diagnosis of PD was not confirmed by the pathological analysis. Additionally, the sample sizes of the PSP and MSA-P groups in this study were relatively small, providing a limitation of this study. Further examination using larger cohorts are needed to clarify the clinical usefulness of DAT-SPECT.

## Methods

### Patients

Subjects were 672 patients, who visited Osaka Medical College Hospital and were examined using DAT-SPECT between March 14, 2014 and December 31, 2018. When patients were regularly given drugs, including selective serotonin reuptake inhibitors, tricyclic antidepressants and opioids, DAT-SPECT was performed after a 2-week drug washout period to reduce a possibility that striatal ^123^I‐Ioflupane accumulation were interfered with these drugs. Of them, 311 patients with PD (158 men) were used for this study (Table 1). The median age was 73 years (IQR 66.0–78.0). The severity of PD was assessed by the HY scale^21^. The median of HY stages was 2 (IQR 1–3). The number of HY stage I, II, III and IV was 88 (38 men, median 70.0 years; IQR 63.0–76.0 years), 106 (57 men, median 73.0 years; IQR 66.0–77.3 years), 89 (53 men, median 75.0 years; IQR 66.5–78.5 years), and 28 (10 men, median 77.5 years; IQR 72.0–82.0 years), respectively. As degenerative parkinsonian syndromes, 33 patients with PSP (18 men, median 74.0 years; IQR 70.5–79.5 years) and 20 patients with MSA-P (12 men, median 71.5 years; IQR 65.8–75.8 years) were used. To focus on the differential diagnosis of degenerative parkinsonian syndromes, we excluded patients with MSA-C from this study. There were no significant differences in the age and percentages of men among the PD, PSP and MSA-P groups. As a control group without parkinsonism, 137 patients were used (62 men, median 76.0 years; IQR 72.0–80.0 years). They included 48 patients with essential tremor (median of average SBR 5.08; IQR 4.08–5.90), 23 patients with orthopaedic diseases (average SBR 4.34; IQR 3.56–6.20), 11 patients with Alzheimer-type dementia (average SBR 5.03; IQR 4.03–5.38), 9 patients with mental diseases (average SBR 4.84; IQR 3.93–6.21), 11 patients with normal aging (average SBR 4.73; IQR 4.28–6.75), 10 patients with endocrine diseases and metabolic diseases (average SBR 5.06; IQR 3.39–6.48), and 25 patients with other reasons (average SBR 5.05; IQR 3.87–6.11). There were no significant differences in average SBRs among these control groups (P = 0.968, Kruskal–Wallis test). Additionally, there was no difference in the values of SBR laterality among the control groups (P = 0.651, Kruskal–Wallis test). These patients underwent DAT-SPECT to rule out degenerative parkinsonian syndromes. Inclusion criteria of this study were: (1) age > 30 years; (2) diagnosis according to the UK Parkinson’s Disease Society Brain Bank diagnostic criteria for PD^22^, National Institute of Neurological Disorders and Stroke-Society for probable PSP^23^ and Gilman’s consensus criteria for probable MSA-P^24^; (3) no evidence of another disease to explain the symptoms. Additionally, the history of unilateral onset was confirmed in most patients with PD. Exclusion criteria were: (1) obvious history of current treatment with psychoactive drugs, such as antiepileptic drugs, tricyclic antidepressant drugs, and serotonin reuptake inhibitors; (2) abnormalities on brain magnetic resonance imaging (MRI), such as diffuse vascular lesions, hydrocephalus, neoplasm, and metal deposition; (3) history of deep brain stimulation.

We retrospectively collected information on age, sex, family history, medication history, neurological symptoms, clinical course, responsiveness to levodopa/decarboxylase inhibitors, presence of cognitive dysfunction, brain MRI, and diagnosis and HY stage from medical records. The diagnosis was not confirmed by the pathological analysis. This study was conducted according to the 2013 Helsinki Declaration, and the Osaka Medical College Ethics Committee approved the study protocol and the need for informed consent was waived because this was a retrospective study and the data were collected without individual patient identifiers (Approval number # 2166).

### Assessment of DAT-SPECT images

At 3–4 h interval after intravenous administration of ^123^I-Ioflupane, head SPECT was performed for 30 min using the GCA 9300R SPECT camera (Canon Medical Systems, Tokyo, Japan). For image analysis, DaTView (Aze Ltd., Tokyo) was used, and values of left and right SBRs were obtained for quantitative evaluation. Region of interest was set to fully cover the striatum. The standard volume of striatum was assumed as 11.2 ml. The area except the striatum was used as the non-specific background. After subtracting the background, the value of SBR was estimated. The evaluation parameters of DAT-SPECT in this study were: (1) average SBR; (2) SBR laterality; and (3) asymmetry index (AI). SBR laterality values were calculated by the following formula: |SBRleft – SBRright|. AI values were calculated by using the following formula: |SBRleft – SBRright| / [(SBRleft + SBRright)/2] × 100^7,19^.

### Statistical analysis

Statistical analysis was performed using the SPSS software program (version 23.0 for MS windows. Chicago, USA). Multiple comparisons above three groups were performed by the Kruskal–Wallis test, and then the Bonferroni correction was performed for pairwise comparisons. The nominal data (sex difference) were analysed by chi-square test. Data were expressed as a median and IQR, and P value < 0.05 was considered statistically significant.

## Data Availability

All data generated or analysed during this study are included in this published article.

## References

[1] Hughes AJ, Daniel SE, Kilford L, Lees AJ (1992). Accuracy of clinical diagnosis of idiopathic Parkinson's disease: A clinico-pathological study of 100 cases. J. Neurol. Neurosurg. Psychiatry.

[2] Postuma RB (2015). MDS clinical diagnostic criteria for Parkinson's disease. Mov. Disord..

[3] Ciliax BJ (1995). The dopamine transporter: Immunochemical characterization and localization in brain. J. Neurosci..

[4] Piggott MA (1998). Nigrostriatal dopaminergic activities in dementia with Lewy bodies in relation to neuroleptic sensitivity: comparisons with Parkinson's disease. Biol. Psychiatry.

[5] Ba F, Martin WR (2015). Dopamine transporter imaging as a diagnostic tool for parkinsonism and related disorders in clinical practice. Parkinsonism Relat. Disord..

[6] Isaias IU (2010). Imaging essential tremor. Mov. Disord..

[7] Zijlmans J (2007). [123I] FP-CIT spect study in vascular parkinsonism and Parkinson's disease. Mov. Disord..

[8] Diaz-Corrales FJ, Sanz-Viedma S, Garcia-Solis D, Escobar-Delgado T, Mir P (2010). Clinical features and 123I-FP-CIT SPECT imaging in drug-induced parkinsonism and Parkinson's disease. Eur. J. Nucl. Med. Mol. Imaging.

[9] Cilia R, Marotta G, Benti R, Pezzoli G, Antonini A (2005). Brain SPECT imaging in multiple system atrophy. J. Neural. Transm. (Vienna).

[10] Kim YJ (2002). Combination of dopamine transporter and D2 receptor SPECT in the diagnostic evaluation of PD, MSA, and PSP. Mov. Disord..

[11] Varrone A, Marek KL, Jennings D, Innis RB, Seibyl JP (2001). [(123)I]beta-CIT SPECT imaging demonstrates reduced density of striatal dopamine transporters in Parkinson's disease and multiple system atrophy. Mov. Disord..

[12] Filippi L (2006). 123I-FP-CIT in progressive supranuclear palsy and in Parkinson's disease: A SPECT semiquantitative study. Nucl. Med. Commun..

[13] Perju-Dumbrava LD (2012). Dopamine transporter imaging in autopsy-confirmed Parkinson's disease and multiple system atrophy. Mov. Disord..

[14] Pirker W (2000). [123I]beta-CIT SPECT in multiple system atrophy, progressive supranuclear palsy, and corticobasal degeneration. Mov. Disord..

[15] Antonini A (2003). 123I-Ioflupane/SPECT binding to striatal dopamine transporter (DAT) uptake in patients with Parkinson's disease, multiple system atrophy, and progressive supranuclear palsy. Neurol. Sci..

[16] Bajaj N, Hauser RA, Grachev ID (2013). Clinical utility of dopamine transporter single photon emission CT (DaT-SPECT) with (123I) ioflupane in diagnosis of parkinsonian syndromes. J. Neurol. Neurosurg. Psychiatry.

[17] Kempster PA, Gibb WR, Stern GM, Lees AJ (1989). Asymmetry of substantia nigra neuronal loss in Parkinson's disease and its relevance to the mechanism of levodopa related motor fluctuations. J. Neurol. Neurosurg. Psychiatry.

[18] Djaldetti R, Ziv I, Melamed E (2006). The mystery of motor asymmetry in Parkinson's disease. Lancet Neurol..

[19] van Dyck CH (2002). Age-related decline in dopamine transporters: analysis of striatal subregions, nonlinear effects, and hemispheric asymmetries. Am. J. Geriatr. Psychiatry.

[20] Matsuda H (2018). Japanese multicenter database of healthy controls for [(123)I]FP-CIT SPECT. Eur. J. Nucl. Med. Mol. Imaging.

[21] Hoehn MM, Yahr MD (1967). Parkinsonism: Onset, progression and mortality. Neurology.

[22] Gibb WR, Lees AJ (1988). The relevance of the Lewy body to the pathogenesis of idiopathic Parkinson's disease. J. Neurol. Neurosurg. Psychiatry.

[23] Litvan I (1996). Clinical research criteria for the diagnosis of progressive supranuclear palsy (Steele-Richardson-Olszewski syndrome): report of the NINDS-SPSP international workshop. Neurology.

[24] Gilman S (2008). Second consensus statement on the diagnosis of multiple system atrophy. Neurology.

